# Cytotoxic Constituents of the Bark of *Hypericum roeperianum* towards Multidrug-Resistant Cancer Cells

**DOI:** 10.1155/2020/4314807

**Published:** 2020-09-25

**Authors:** Michel-Gael F. Guefack, Francois Damen, Armelle T. Mbaveng, Simplice Beaudelaire Tankeo, Gabin T. M. Bitchagno, İlhami Çelik, James D. Simo Mpetga, Victor Kuete

**Affiliations:** ^1^Department of Biochemistry, Faculty of Science, University of Dschang, P.O. Box 67, Dschang, Cameroon; ^2^Department of Chemistry, Faculty of Science, University of Dschang, P.O. Box 67, Dschang, Cameroon; ^3^Department of Chemistry, Faculty of Science, Eskisehir Technical University, Eskisehir 26470, Turkey

## Abstract

The global cancer burden remains a serious concern with the alarming incidence of one in eight men and one in eleven women dying in developing countries. This situation is aggravated by the multidrug resistance (MDR) of cancer cells that hampers chemotherapy. In this study, the cytotoxicity of the methanol extract (HRB), fractions (HRBa, HRBb, and HRBa1-5), and compounds from the bark of *Hypericum roeperianum* (HRB) was evaluated towards a panel of 9 cancer cell lines. The mode of action of the HRB and trichadonic acid (**1**) was also studied. Column chromatography was applied to isolate the constituents of HRB. The cytotoxicity of botanicals and phytochemicals was evaluated by the resazurin reduction assay (RRA). Caspase-Glo assay was used to evaluate the activity of caspases, and reactive oxygen species (ROS) (H_2_DCFH-DA) were assessed by flow cytometry. Phytochemicals isolated from HRB were trichadonic acid (**1**), fridelan-3-one (**2**), 2-hydroxy-5-methoxyxanthone (**3**), norathyriol (**4**), 1,3,5,6-tetrahydroxyxanthone (**5**), betulinic acid (**6**), 3′-hydroxymethyl-2′-(4″-hydroxy-3″,5″-dimethoxyphenyl)-5′,6′:5,6-(6,8-dihydroxyxanthone)-1′,4′-dioxane (**7**), and 3′-hydroxymethyl-2′-(4″-hydroxy-3″,5″-dimethoxyphenyl)-5′,6′:5,6-(xanthone)-1′,4′-dioxane (**8**). Botanicals HRB, HRBa, HRBa2-4, HRBb, and doxorubicin displayed cytotoxic effects towards the 9 tested cancer cell lines. The recorded IC_50_ values ranged from 11.43 *µ*g/mL (against the P-glycoprotein (gp)-overexpressing CEM/ADR5000 leukemia cells) to 26.75 *µ*g/mL (against HCT116 (p53^+/+^) colon adenocarcinoma cells) for the crude extract HRB. Compounds **1**, **5**, and doxorubicin displayed cytotoxic effects towards the 9 tested cancer cell lines with IC_50_ values varying from 14.44 *µ*M (against CCRF-CEM leukemia cells) to 44.20 *µ*M (against the resistant HCT116 (p53^−/−^) cells) for **1** and from 38.46 *µ*M (against CEM/ADR5000 cells) to 112.27 *µ*M (against the resistant HCT116 (p53^−/−^) cells) for **5**. HRB and compound **1** induced apoptosis in CCRF-CEM cells. The apoptotic process was mediated by enhanced ROS production for HRB or *via* caspases activation and enhanced ROS production for compound **1**. This study demonstrated that *Hypericum roeperianum* is a potential source of cytotoxic phytochemicals such as trichadonic acid and could be further exploited in cancer chemotherapy.

## 1. Introduction

Cancer continues to be a global threat, appearing as the second leading cause of death globally, with estimated 9.6 million deaths representing one in six deaths and with an estimated five-year prevalence of 43.8 million people [1]. Multidrug resistance (MDR) of cancer cells is a serious concern in chemotherapy. It is responsible for many therapeutic failures and high burdens globally, in patients suffering from cancer [2, 3]. Any modern protocol for new cytotoxic drug discovery today should integrate the ability of neoplastic cells to rapidly develop resistant phenotypes. Thus, resistant cell lines should be integrated into the cell panel used for the discovery of more efficient substances. The present work has taken this into account and involves several models of MDR cancer cell lines such as the colon adenocarcinoma with p53 knockout phenotype, the leukemia cells with ATP-binding cassette (ABC)-transporter-overexpressing MDR-mediating-P-glycoprotein (P-gp; ABCB1/MDR1), the breast cancer bearing resistance protein (ABCG2/BCRP), and the transfectant glioblastoma multiforme harboring a mutation-activated EGFR gene (ΔEGFR). The effectiveness of natural products in the fight against cancer has been largely demonstrated [4]. Some clinically established cytotoxic drugs such as camptothecin, paclitaxel, vinblastine, or vincristine are naturally occurring compounds [4–6]. In addition, numerous botanicals and phytochemicals derived from African medicinal plants have been found active against MDR cancer cell lines [7, 8]. Some of such prominent phytochemicals include terpenoids: salvimulticanol and candesalvone B methyl ester [9], epunctanone [10], and ardisiacrispin B [11], phenolics: 2-acetyl-7-methoxynaphtho [2,3-b]furan-4,9-quinone [12], 6*α*-hydroxyphaseollidin [13], licoagrochalcone A [14], 7-dihydroxy-4′-methoxy-6,8-diprenylisoflavone, and 7,7″-di-*O*-methylchamaejasmin [15], and alkaloids: 1,3-dimethoxy-10-methylacridone [16], isotetrandrine [17], and ungeremine [18]. However, more hit compounds should be identified to increase our arsenal of cytotoxic compounds and to secure better the chances of later obtaining new clinically usable molecules. The present study was, therefore, designed to assess the cytotoxicity of botanicals and phytochemicals from the bark of *Hypericum roeperianum* Schimp. p. ex A. Rich (Guttiferae). The modes of action of compound **1**, such as its effects on cell cycle distribution and induction of apoptosis, on caspases activation, and on the production of reactive oxygen species (ROS), were also investigated. *Hypericum roeperianum* is a shrub or small tree growing in the tropical part of central, eastern, and southern tropical Africa, locally used alone or in association with various plants in the treatment of female sterility [19], as antiabortifacients [20] and as antifungal remedies [21]. Previous phytochemical investigations of this plant led to the isolation of a polyketide, 4-methoxy-3-(2-methylbut-3-en-2-yl)-6-phenyl-2*H*-pyran-2-one, xanthones: 1,5-dihydroxy-6-methoxyxanthone, 2-hydroxy-5-methoxyxanthone and 1,4,6,7-tetrahydroxyxanthone, and the xanthonolignoids: 8,10-dihydroxy-3-(4hydroxy-3,5-dimethoxyphenyl-2-(hydroxymethyl)-2, 3-dihydro-[1, 4]dioxino[2,3-c]xanthen-7-one and 8-hydroxy-10-methoxy-3-(4-hydroxy-3,5-dimethoxyphenyl-2-(hydroxymethyl)-2,3-dihydro-[1,4]dioxino[2,3-c]xanthen-7-one from the bark [22] and 10 other xanthones from the roots, namely, 5-*O*-methyl-2-deprenylrheediaxanthone B, 5-*O*-methylisojacareubin, 5-*O*-demethylpaxanthonin, roeperanone, 2-hydroxyxanthone, 5-hydroxy-2-methoxyxanthone, 1,5-dihydroxy-2-methoxyxanthone, 2-deprenyl rheediaxanthone B, isojacareubin, and calycinoxanthone D [23]. The cytotoxicity of botanicals from the bark of *Hypericum roeperianum* is being reported for the first time.

## 2. Material and Methods

### 2.1. Chemicals

Doxorubicin (98.0% purity) from Sigma-Aldrich (Munich, Germany) was obtained from the Johannes Gutenberg University Medical Center (Mainz, Germany). Geneticin >98% (used at 800 ng/mL and 400 *µ*g/mL) in culture media to maintain the features of MDA-MB-231-*BCRP,* U87MG.Δ*EGFR*, and HCT116 (*p53*^−^/^−^), respectively, was obtained from Sigma-Aldrich and stored at 72.18 mM. Hydrogen peroxide (H_2_O_2_) and valinomycin (at 1 mg/mL) were provided by Sigma-Aldrich (Taufkirchen, Germany).

### 2.2. Plant Material and Extraction

The bark of *Hypericum roeperianum* Schimp. p. ex A. Rich (Guttiferae) was collected in Bangang Wabane (South West Region of Cameroon) in October 2018. No permission was necessary for sample's collection. The identification of the plant was carried out by Dr. Tchiengue Barthelemy at the Cameroon National Herbarium (Yaoundé) where a voucher specimen was deposited under the number 24584/SRF/Cam. Dried barks of the plant (3.0 kg) were powdered and extracted with methanol (MeOH; 3 × 15 L) for 72 h at room temperature to afford a crude extract (HRB; 150.0 g) after filtration with Whatman paper no.1 and evaporation in vacuum, under reduced pressure. A portion of the resulting extract (140.0 g) was, then, exhausted in ethyl acetate to yield 65.0 g of the ethyl acetate extract (EtOAc) (HRBa) and the residue (HRBb; 75 g).

### 2.3. Fractionation and Purification of the Bark Extract of *Hypericum roeperianum*

Part of the ethyl acetate extract (EtOAc; 60.0 g) was submitted to silica gel flash chromatography using hexane-EtOAc and, then, EtOAc-MeOH mixtures of increasing polarities. Eighty fractions (frs) of 500 mL each were collected as follows: hexane 100% (sub-frs 1–3), hexane-EtOAc 90 : 10 (sub-frs 4–12), hexane-EtOAc 80 : 20 (sub-frs 13–18), hexane-EtOAc 70 : 30 (sub-frs 19–22), hexane-EtOAc 60 : 40 (sub-frs 23–27), hexane-EtOAc 50 : 50 (sub-frs 28–37), hexane-EtOAc 30 : 70(sub-frs 38–43), AcOEt 100% (sub-frs 44–52), EtOAc-MeOH 95 : 5 (sub-frs 53–57), EtOAc-MeOH 90 : 10 (sub-frs 58–62), EtOAc-MeOH 80 : 20 (sub-frs 63–69), and MeOH 100% (sub-frs 70–80). Based on their analytical thin-layer chromatography (TLC) profiles, these fractions were pooled into five fractions (frs) as follows: HRBa1 (Sub-frs 1–15, 8.0 g), HRBa2 (Sub-frs 16–25, 15.0 g), HRBa3 (Sub-frs 28–38, 9.5 g), HRBa4 (Sub-frs 39–68, 12.5 g), and HRBa5 (Sub-frs 69–80, 13.0 g).

Dry fraction HRBa2 (15.0 g) was dissolved in methanol affording a nonsoluble powder which was, then, filtered to give compound **1** (15 mg). The filtrate was subjected to silica gel column chromatography using hexane-AcOEt mixtures of increasing polarities as elution solvents. Sixty-five sub-frs of 150 mL each were collected as follows: Hex 100% (1–3), Hex-AcOEt 90:10 (Sub-frs 4–9), Hex-AcOEt 80 : 20 (Sub-frs 10–15), Hex-AcOEt 70 : 30 (Sub-frs 16–19), Hex-AcOEt 60 : 40 (Sub-frs 20–35), Hex-AcOEt 50 : 50 (Sub-frs 36–42), Hex-AcOEt 40 : 60 (Sub-frs 43–50), AcOEt 100% (Sub-frs 51–55), AcOEt-MeOH 90 : 10 (Sub-frs 56–60), and MeOH 100% (Sub-frs 61–65). Sub-frs 6–9 yielded compound **2** (15.0 mg) as a white powder. Sub-frs EC23-29 yielded compound **3** (12.0 mg) as a yellow powder.

HRBa3 (9.5 g) was subjected to silica gel column chromatography using Hex-AcOEt mixtures of increasing polarities as elution solvents. Seventy-five sub-frs of 150 mL each were collected as follows: hexane 100% (sub-frs 1–4), Hex-AcOEt 90 : 10 (sub-frs 5–9), Hex-AcOEt 80 : 20 (sub-frs 9–16), Hex-AcOEt 70 : 30 (sub-frs 17–23), Hex-AcOEt 60 : 40 (sub-frs 24–35), Hex-AcOEt 50 : 50 (sub-frs 36–42), Hex-AcOEt 40 : 60 (sub-frs 43–47), AcOEt 100% (sub-frs 48–57), AcOEt-MeOH 90 : 10 (sub-frs 58–63), AcOEt-MeOH 80 : 20 (sub-frs 64–70), and MeOH 100% (sub-frs 71–75). Sub-frs 22–25 yielded compound **4** (18.0 mg) as a green-yellowish powder. Sub-frs 29–35 yielded compound **5** as a yellow powder (15.0 mg).

HRBa4 (12.5 g) was subjected to silica gel column chromatography using CH_2_Cl_2_-MeOH mixtures of increasing polarities as elution solvents. Fifty sub-frs of 150 mL each were collected as follows: CH_2_Cl_2_ 100% (sub-frs 1–4), CH_2_Cl_2_-MeOH 95 : 5 (sub-frs 5–13), CH_2_Cl_2_-MeOH 90 : 10 (sub-frs 14–25), CH_2_Cl_2_-MeOH 85 : 15 (sub-frs 26–35), CH_2_Cl_2_-MeOH 80 : 20 (sub-frs 36–42), and MeOH 100% (subfrs 43–50). Sub-frs 6–11 yielded compound **6** (40.0 mg) as a white powder. Sub-frs 13–15 yielded compound **7** (12.0 mg) as a yellow powder. Sub-frs 19–23 yielded compound **8** (14.0 mg) as a yellow powder.

### 2.4. Cell Cultures

Cell lines used in this work included drug-sensitive and drug-resistant phenotypes of earlier reported origin. They were all provided by Prof. Dr. Thomas Efferth from his cell lines collection; they have being used in cytotoxicity screening by our team for a decade [12–21]. These include two hematological cancer cell lines, namely, the drug-sensitive CCRF-CEM leukemia cell line and its multidrug-resistant P-gp-over-expressing subline CEM/ADR5000 cells [24–26] and nine carcinoma cell lines, namely, U87.MG glioblastoma cell line and its EGFR-transfected U87.MGΔ*EGFR* subline, HCT116 (*p53*^*+/+*^) colon cancer cell line and its knockout clone HCT116 (*p53*^−^/^−^), and MDA-MB-231-pcDNA3 breast cancer cell line and its *BCRP*-transfected multidrug-resistant MDA-MB-231-*BCRP* clone 23 cell line [27], as well as the normal AML12 hepatocytes, used to compare with HepG2 liver cancer cells [13].

### 2.5. Resazurin Reduction Assay (RRA) for Cell Growth Evaluation

The RRA was applied to evaluate the cytotoxicity of botanicals, the isolated phytochemicals (**1–5, 7**, and **8**), and doxorubicin on the cell growth as reported earlier [18, 28]. Cells treated with various samples at different concentrations were incubated for 72 h in humidified 5% CO_2_ atmosphere at 37°C. Cells were further coloured with resazurin and incubated for 1–2 h; the fluorescence was further measured with an Infinite M2000 Pro^™^ plate reader (Tecan, Crailsheim, Germany) at 544 nm as the excitation wavelength and 590 nm as the emission wavelength. The IC_50_ values represented the concentrations of the sample required to inhibit 50% of cell proliferation and were calculated from a calibration curve by linear regression using Microsoft Excel 2007 [29].

### 2.6. Flow Cytometric Evaluation of Cell Cycle Distribution and Apoptotic Cells

Various concentrations of botanical HRB, phytochemical **1**, and doxorubicin or DMSO (solvent control) were used to treat CCRF-CEM cells (1 × 10^6^ cells). Cells were further incubated for 24 h in humidified 5% CO_2_ atmosphere at 37°C and analyzed using a BD Accury C6 Flow Cytometer (BD Biosciences, Heidelberg, Germany) by measuring the propidium iodide fluorescence of individual nucleus, as described earlier [10, 11]. Experiments were conducted thrice independently with three parallel measurements.

### 2.7. Assessment of Apoptosis by Annexin V/PI Staining

The CCRF-CEM cells (1 × 10^6^; 1 ml) were also treated with HRB, compound **1** and doxorubicin for 24 h (in humidified 5% CO_2_ atmosphere at 37°C), and apoptosis was further assessed by flow cytometry using the flouresceinisothiocynate- (FITC-) conjugated annexin V/PI assay kit (eBioscience^™^Annexin V; Invitogen, San Diego, USA), as previously published [10, 11]. Briefly, treated cells were centrifuged at 1200 rpm for 5 min, then washed twice with ice-cold PBS, resuspended in 500 *µ*l binding buffer, and stained with 5 *µ*l FITC-conjugated annexin V (10 mg/mL) and 10 *µ*l PI (50 mg/ml). After 15 min incubation at room temperature (RT) in the dark, cells were analyzed using a BD Accury C6 Flow Cytometer (BD Biosciences). Cells stained with only annexin V were evaluated as being in early apoptosis. Cells stained with both annexin V and propidium iodide were evaluated as being in late apoptosis or in a necrotic stage.

### 2.8. Evaluation of Caspases Activities Using Caspase-Glo 3/7, Caspase-Glo 8, and Caspase-Glo 9

Different concentrations of HRB and compound **1** were used to treat CCRF-CEM cells for 6 h. The activities of caspases were determined using Caspase-Glo 3/7, Caspase-Glo 8, and Caspase-Glo 9 Assay kits (Promega, Mannheim, Germany) by measuring the luminescence using an Infinite M2000 ProTM plate reader (Tecan), as reported previously [13].

### 2.9. Evaluation of Reactive Oxygen Species (ROS) Production

Various concentrations of HRB and triterpenoid **1** were used to treat CCRF-CEM cells (1 × 10^6^ cells); DMSO (solvent control); or hydrogen peroxide (H_2_O_2_; positive control). After 24 h incubation in humidified 5% CO_2_ atmosphere at 37°C, the production of ROS was evaluated using 2′,7′-dichlorodihydrofluorescein diacetate (H_2_DCFH-DA) (Sigma-Aldrich) staining, as described earlier [30–32].

### 2.10. Statistics

Statistical analyses were performed with Graph pad prism 5 software. Representative data from three independent experiments are shown as mean value ± S.E.M. One-way Analysis Variance (ANOVA) followed by post hoc Tukey's test was used to determine the significance of the difference between mean values relative to the control. The *p* value was calculated to determine significant differences (*p* value < 0.05).

## 3. Results

### 3.1. Phytochemistry

**Figure 1:**
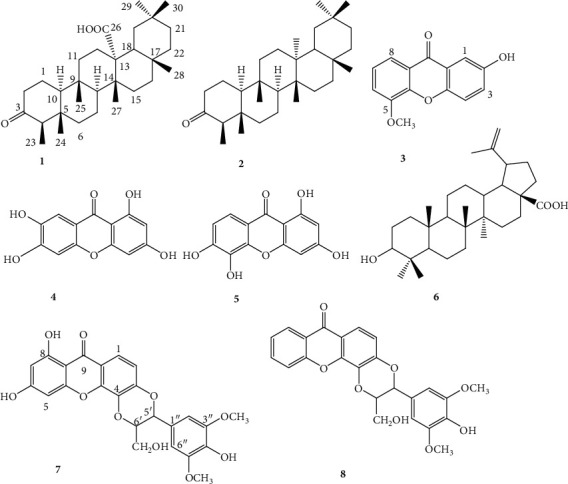
Chemical structures of phytochemicals isolated from the bark methanol extract of *Hypericum roeperianum*. **1**: Trichadonic acid; **2**: fridelan-3-one; **3**: 2-hydroxy-5-methoxyxanthone; **4** : 1,3,6,7-tetrahydroxyxanthone or norathyriol; **5** : 1,3,5,6-tetrahydroxyxanthone; **6**: betulenic acid; and **7** : 3′-hydroxymethyl-2′-(4″-hydroxy-3″,5″-dimethoxyphenyl)-5′,6′:5,6-(6,8-dihydroxyxanthone)-1′,4′-dioxane; 8 : 3′-hydroxymethyl-2′-(4″-hydroxy-3″,5″-dimethoxyphenyl)-5′,6′:5,6-(xanthone)-1′,4′-dioxane.

The chemical structures of the isolated phytochemicals were determined by exploiting the physical, mass spectra, and NMR data, followed by direct comparison of these data with those of similar reported compounds in the literature. Compounds were identified as trichadonic acid C_30_H_48_O_3_ (**1**; white amorphous powder; *m/z* 456) [33], fridelan-3-one C_30_H_50_O (**2**; white powder; m.p. 258^o^C; *m/z* 426) [33], 2-hydroxy-5-methoxyxanthone C_14_H_1*0*_O_4_ (**3**; yellow amorphous powder; *m/z* 242) [34], 1,3,6,7-tetrahydroxyxanthone or norathyriol C_13_H_8_O_6_ (**4**; green-yellowish powder; m.p. 271^o^C; *m/z* 260) [35], 1,3,5,6-tetrahydroxyxanthone C_13_H_8_O_6_ (**5**; yellow powder; m.p. 136^o^C; *m/z* 260) [36], betulenic acid C_30_H_48_O_3_ (**6**; white powder; m.p. 318^o^C; *m/z* 456) [33], 3′-hydroxymethyl-2′-(4″-hydroxy-3″,5″-dimethoxyphenyl)-5′,6′:5,6-(6,8-dihydroxyxanthone)-1′,4′-dioxane C_24_H_20_O_8_ (**7**; yellow powder; m.p. 264^o^C; *m/z* 436) [37, 38], and 3′-hydroxymethyl-2′-(4″-hydroxy-3″,5″-dimethoxyphenyl)-5′,6′:5,6-(xanthone)-1′,4′-dioxane C_24__20_ O_10_ (**8**; yellow amorphous powder; *m/z* 468) [37] (Figure 1). The 1D NMR spectra of these compounds are provided as Supplementary Materials.

### 3.2. Cytotoxicity of Phytochemicals and Doxorubicin

The cytotoxicity of crude extracts, fractions, and phytochemicals **1–5**, **7**, **8**, and doxorubicin was investigated using RRA towards 9 cancer cell lines and normal AML12 hepatocytes (Tables 1 and 2). The degree of resistance (D.R.) of the tested samples was determined as the ratio of the IC_50_ value of the resistant cell line divided by that of the corresponding parental sensitive cell line (Tables 1 and 2). Collateral sensitivity or hypersensitivity was deduced if the D.R. was below 1 while normal sensitivity was defined as a D.R. of 1 or around 1; cross resistance was considered as a D.R. above 1. The selectivity index (S.I.) was also calculated as the ratio of the IC_50_ value in normal AML12 hepatocytes by the corresponding values in HepG2 hepatocarcinoma cells (Tables 1 and 2).

The obtained IC_50_ values ranged from 11.43 *µ*g/mL (against the P-gp-overexpressing CEM/ADR5000 leukemia cells) to 26.75 *µ*g/mL (against HCT116 (p53^+/+^) colon adenocarcinoma cells) for the crude extract HRB, from 15.65 *µ*g/mL (against CEM/ADR5000 leukemia cells) to 41.17 *µ*g/mL (against HCT116 (p53^+/+^) cells) for HRBa, from 13.92 *µ*g/mL (against U87MG.ΔEGFR glioblastoma cells) to 33.44 *µ*g/mL (against HCT116 (p53^+/+^) cells) for HRBa2, from 16.13 *µ*g/mL (against U87MG glioblastoma cells) to 33.63 *µ*g/mL (against HCT116 (p53^−/−^) cells) for HRBa3, from 10.52 *µ*g/mL (against U87MG.ΔEGFR cells) to 28.43 *µ*g/mL (against HCT116 (p53^+/+^) cells) for HRBa4, and from 28.30 *µ*g/mL (against U87MG.ΔEGFR cells) to 69.48 *µ*g/mL (against HepG2 cells) for HRBb (Table 1). Fractions HRBa1 and HRBa5 had selective activities (Table 1).

Triterpenoid **1**, xanthone **5**, and doxorubicin displayed cytotoxic effects towards the 9 tested cancer cell lines with IC_50_ values ranging from 14.44 *µ*M (against CCRF-CEM cells) to 44.20 *µ*M (against the resistant HCT116 (p53^−/−^) cells) for **1**, from 38.46 *µ*M (against CEM/ADR5000 cells) to 112.27 *µ*M (against HCT116 (p53^−/−^) cells) for **5**, and from 0.02 *µ*M (against CCRF-CEM cells) to 122.96 *µ*M (against CEM/ADR5000 cells) for doxorubicin (Table 2). Xanthones **3** and **4**, as well as xantholignans **7** and **8**, had selective cytotoxic effects (Tables 2).

Collateral sensitivity (D.R. below 1) of CEM/ADR5000 cells, *BCRP*-expressing MDA-MB-231 cells, and HCT116 (p53^−/−^) cells to the mother botanical, HRB compared to their sensitive counterparts CCRF-CEM cells, MDA-MB-231 cells, and HCT116 (p53^+/+^) cells, respectively, was observed. Hypersensitivity of all resistant cell lines to fraction HRBa4 and HRBb compared to their sensitive parental cell lines was also recorded (Table 1). Collateral sensitivity of *BCRP*-expressing MDA-MB-231 cells and U87MG.ΔEGFR cells to phytochemicals **1**, **3–5**, and **8** compared to their sensitive counterparts MDA-MB-231 cells and U87MG cells, respectively, was also observed (Table 2). Concerning the most active compound **1**, a little cross resistance of HCT116 (p53^−/−^) cells compared to their sensitive counterparts HCT116 (p53^+/+^) was observed with a D.R. of 2.55; however, this value was lower than that obtained with doxorubicin (D.R. of 3.73) (Table 2). Apart against HRBb, the S.I. of all samples was above 2 in HepG2 as compared with normal AML12 hepatocytes (Table 1). Compounds **1**, **3**, **5**, **8**, and doxorubicin had an S.I. above 2 in HepG2 as compared with normal AML12 hepatocytes (Table 1). Regarding the recorded IC_50_ values, trichadonic acid (**1**) had the best activity and was consequently selected, together with the crude extract, HRB, for further mechanistic studies.

### 3.3. Cell Cycle Distribution and Apoptosis

**Figure 2:**
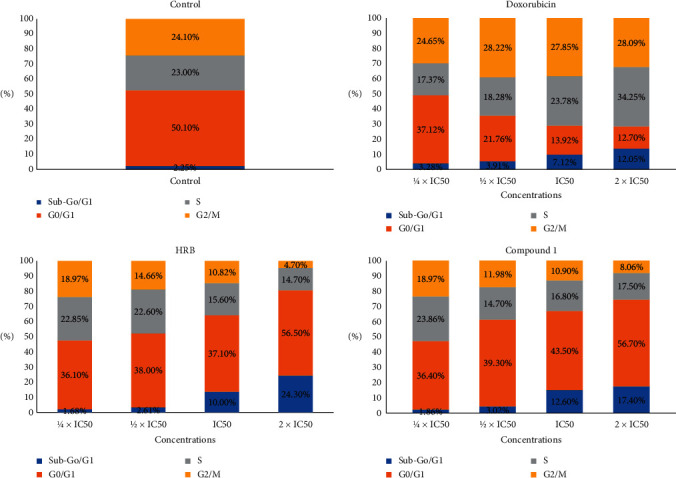
Distribution of the CCRF-CEM cells cycle after treatment with the crude extract (HRB), compound **1** (trichadonic acid), and doxorubicin. IC_50_ values were 14.44 *µ*M for trichadonic acid and 0.02 *µ*M for doxorubicin.

**Figure 3:**
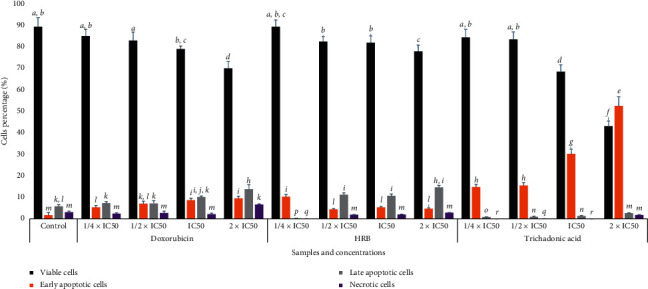
Apoptosis induced by the crude extract (HRB), trichadonic acid, and doxorubicin after 24 h on CCRF-CEM leukemia cells as determined by annexin V/PI assay. Apoptosis was assessed by flow cytometry after annexin V-PI double staining. IC_50_ values were 13.71 *µ*g/mL for HRB, 14.44 *µ*M for trichadonic acid, and 0.02 *µ*M for doxorubicin. Mean values ± SD of three independent experiments is shown. Data with different superscript letters are significantly different (*p* < 0.05).

The crude extract (HRB) and triterpenoid **1**, as well as doxorubicin, caused dose-dependent alteration of CCRF-CEM cells' cycle distribution after 24 h treatment (Figure 2). Figure 2 shows that HRB and trichadonic acid (**1**) induced increase of cells in the sub-G0/*G*1 phase, varying from 1.68% (1/4 × IC_50_) to 24.30% (2 × IC_50_) for HRB and from 1.86% (1/4 × IC_50_) to 17.40% (2 × IC_50_) for compound **1**, while doxorubicin induced increase in the range of 3.28% (1/4 × IC_50_) to 12.05% (2 × IC_50_). This suggests that HRB and compound **1** induced apoptosis in CCRF-CEM cells. Increase of cells in the Go/*G*1 phase also suggests that both HRB and trichadonic acid caused cycle arrest in this phase; meanwhile, doxorubicin induced S and G2/M phase arrest (Figure 2). A concentration-dependent induction of apoptosis by HRB and compound **1**, as well as doxorubicin, was further confirmed by annexin V/PI staining (Figure 3). At 2 × IC_50_ for example, HRB significantly (*p* < 0.05) induced late apoptosis (Q2-UR) while compound **1** significantly (*p* < 0.05) induced early apoptosis with, respectively, 14.6% annexin V (+)/PI (−) and 52.5% annexin V (+)/PI (+) cells (Figure 3).

### 3.4. Activation of Caspases and Production of ROS

**Figure 4:**
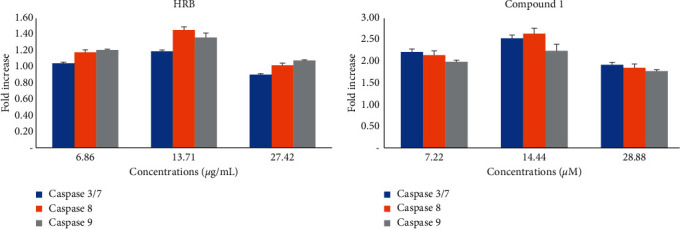
Caspases activities in CCRF-CEM cells treated with the crude extract (HRB) and trichadonic acid for 6 h IC_50_ of HRB: 13.71 *µ*g/mL and trichadonic acid: 14.44 *µ*M; (A) caspase activity is expressed as percentage (%) compared to untreated cells. Mean ± SD of three independent experiments is shown.

In the presence of the tested samples, the activity of caspases in CCRF-CEM cells increased by 1.18-fold, 1.45-fold, and 1.35-fold for HRB and by 2.52-fold, 2.62-fold, and 2.23-fold for trichadonic acid (**1**), respectively, for caspases 3/7, 8, and 9 (Figure 4).

**Figure 5:**
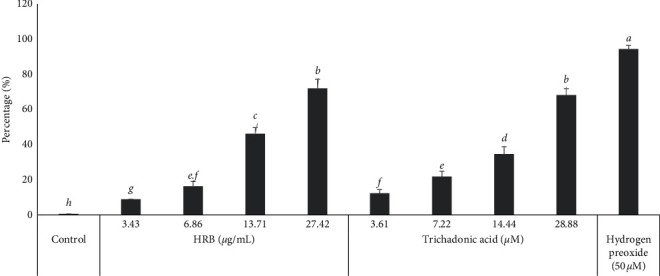
Effects of the crude extract (HRB), trichadonic acid, and hydrogen peroxide (H_2_O_2_) on the production of reactive oxygen species (ROS) in CCRF-CEM cells treated for 24 h. Mean ± SD of three independent experiments is shown. Data with different superscript letters are significantly different (*p* < 0.05).

The production of reactive oxygen species (ROS) in CCRF-CEM cells treated with HRB, triterpenoid **1**, H_2_O_2_ (positive control), or DMSO was analyzed, and the results are depicted in Figure 5. The crude extract HRB significantly (*p* < 0.05) induced increase of ROS production in a range of 8.98% (1/4 × IC_50_) to 71.92% (2 × IC_50_); compound **1** also significantly (*p* < 0.05) induced increase of ROS production in a range of 12.35% (7.22 *µ*M) to 68.12% (57.76 *µ*M). The reference compound, H_2_O_2_, increased the ROS levels to 94.30% at 50 *µ*M, while ROS production in nontreated cells was 0.6%.

## 4. Discussion

Taking into account the rapid development of resistance by cancer cell lines, the use of MDR phenotypes when screening phytochemicals is an interesting approach. Collateral or normal sensitivity (D.R. below or equal to 1) of MDR cells to phytochemicals combined to their good cytotoxicity could be better criteria to select substances for clinical studies. In the present work, four MDR cells lines, CEM/ADR5000 cells, MDA-MB-231-*BCRP* cells, HCT116 (*p53*^−^/^−^) cells, and U87.MGΔ*EGFR* cells, were used, and their susceptibilities to isolated phytochemicals were compared with those of their parental sensitive counterparts, CCRF-CEM cells, MDA-MB-231 cells, HCT116 (*p53*^*+/+*^) cells, and U87.MG cells, respectively (Tables 1 and 2). Interestingly, collateral sensitivity of CEM/ADR5000 cells, *BCRP*-expressing MDA-MB-231 cells, and HCT116 (p53^−/−^) cells to HRB was achieved, as well as the hypersensitivity of all resistant cell lines to fraction HRBa4 and HRBb compared to their sensitive parental cell lines (Table 1). Collateral sensitivity of *BCRP*-expressing MDA-MB-231 cells and U87MG.ΔEGFR cells to phytochemicals **1**, **3–5**, and **8** was observed, suggesting that they might be useful to fight drug resistance in breast cancer and glioblastoma (Table 2). This clearly indicates that these botanicals and phytochemicals can be exploited in the fight against recalcitrant cancers. The IC_50_ values below 20 *μ*g/mL or below 10 *μ*M after incubation between 48 and 72 h have been set for promising cytotoxic botanicals and phytochemicals, respectively [39, 40]. Importantly, IC_50_ values below 20 *µ*g/mL were obtained with HRB against 8/9 tested cancer cells lines, HRBa2 and HRBa4 against 7/9 cancer cell lines, HRBa against 4/9 cancer cell lines, and HRBa3 against 3/9 cell lines (Table 1). It can, therefore, be confirmed that these botanicals are interesting cytotoxic agents. However, IC_50_ values below the established threshold were not achieved with phytochemicals, though terpenoid **1** and xanthone **5** had cytotoxic effects towards the 9 tested cancer cell lines. However, their good selectivity indexes still suggest that they can still be good candidates to tackle cancers, especially when drug resistance is observed. To the best of our knowledge, the cytotoxicity of the crude extract and compounds **1**, **2**, **3**, **7**, and **8** on the studied cell lines is being reported, herein, for the first time. Betulinic acid (**6**) is a well-known cytotoxic compound and has previously been found active towards the cancer cell lines tested in the present work, with IC_50_ values ranging from 7.65 *µ*M (in CEM-ADR5000 cells) to 44.17 *µ*M (in HepG2 cells) [41]. Although it was not further tested, herein, compound **6** can be ranked amongst the best active principles of *Hypericum roeperianum.* Also, the cytotoxicity of norathyriol (**4**) in JB6 P+ mouse skin epidermal cells was reported [42]. 1,3,5,6-Tetrahydroxyxanthone (**5**) had low cytotoxic effects against K562 leukemia cells with 12.98 *µ*g/mL (49.92 *µ*M) [43].

Apoptosis is a programmed cell death and is also the most investigated mechanism of action of antiproliferative drugs. In this study, it was found that both HRB and compound **1** induced apoptosis in CCRF-CEM cells with cell cycle arrest in the Go/*G*1 phase (Figures 2 and 3). Modulation of caspases activities is one of the events observed in the apoptotic process in cancer cell lines [44], making these enzymes a target for cytotoxic drug discovery [8, 45]. However, no significant increase in the activity of initiator caspases (caspases 8 and 9) or in that of the activator caspases (caspase 3/7) was observed (Figure 4). Phytochemical **1** induced 2.52-fold, 2.62-fold, and 2.23-fold increase of the activity of caspases 3/7, 8, and 9, respectively (Figure 4), suggesting that this molecule is a caspase modulator. Botanical HRB and compound **1** were also shown to induce increase of ROS by up to 71.92% and 68.12%, respectively (2 × IC_50_; Figure 5); this is an indication that one of the modes of action of this triterpenoid also includes the enhancement of ROS production in cancer cells.

Regarding the structure-activity relationship, it appears that pentacyclic triterpene **1** is different from **2** by the presence of the carboxyl group (-COOH) in C-13 (Figure 1); the presence of this −COOH group significantly enhanced the cytotoxic activity of triterpene **1**, with IC_50_ values ranging from 14.44 *µ*M to 44.20 *µ*M in cancer cells tested whilst no IC_50_ value at up to 100 *µ*M was recorded with triterpene **2** (Table 1). Betulic acid, another pentacyclic triterpene with–COOH in C-17, previously displayed good cytotoxicity against all cancer cell lines tested in this work [41], illustrating the importance of the carboxyl function in the cytotoxicity of pentacyclic triterpenes. Concerning xanthones, though **5** was active in all tested cancer cell lines, **3-5** displayed moderate activities (Table 1). It was previously shown that additional hetrocycle in xanthone combined to prenylation improved the cytotoxicity of xanthones, with cudraxanthone I (additional hetrocycle combined to -C-8-prenylation) displaying significant cytotoxic effects (IC_50_ value below 10 *µ*M) against all cancer cell lines tested in the present study [46]. This observation is also confirmed with another prenylated xanthone bearing additional herocycle, xanthone V1 [47]. The difference between the two xanthonolignoids **7** and **8** is the presence of two hydroxyl (-OH) groups in C-6 and C-7 (Compound **8**) (Figure 1). This difference seems to influence the selectivity, as compound **8** was active against HCT116 (*p53*^*+/+*^) cells, with an IC_50_ value of 37.79 *µ*M compared to the IC_50_ value above 91.74 *µ*M obtained for compound **7** (against the same cell line (Table 1).

## 5. Conclusions

The present work demonstrated that *Hypericum roeperianum* is a source of cytotoxic compounds. Triterpenoids such as trichadonic acid (**1**) and betulinic acid (**6**), xonthones (2-hydroxy-5-methoxyxanthone (**3**), norathyriol (**4**), and 1,3,5,6-tetrahydroxyxanthone (**5**)), and xantholignoids (3′-hydroxymethyl-2′-(4″-hydroxy-3″,5″-dimethoxyphenyl)-5′,6′:5,6-(6,8-dihydroxyxanthone)-1′,4′-dioxane (**7**) and 3′-hydroxymethyl-2′-(4″-hydroxy-3″,5″-dimethoxyphenyl)-5′,6′:5,6-(xanthone)-1′,4′-dioxane (**8**)) are amongst the active constituents of this plant. Trichadonic acid (**1**) induced apoptosis in CCRF-CEM leukemia cells, through caspases activation and enhancement of ROS production. The crude extract, HRB, also induced apoptosis in CCRF-CEM cells, mediated by enhancement of ROS production. These compounds can potentially be useful in the fight against recalcitrant cancers.

## Figures and Tables

**Table 1:** Recorded IC_50_ values after 72 h incubation of drug-sensitive and MDR cancer cells lines with botanicals from the bark of *Hypericum roeperianum*.

Cell lines	Samples, IC_50_ values in *µ*g/mL, and degrees of resistance^*∗*^ or selectivity index^*∗∗*^							
	HRB	HRBa	HRBa1	HRBa2	HRBa3	HRBa4	HRBa5	HRBb
CCRF-CEM	**13.71** **±** **0.26**	**15.65** **±** **1.18**	46.42 ± 1.98	**17.87** **±** **1.53**	**16.81** **±** **4.02**	**16.27** **±** **4.28**	57.15 ± 3.76	48.76 ± 2.83
CEM/ADR5000	**11.43** **±** **0.88**	**18.56** **±** **1.74**	>80	**20.18** **±** **1.76**	23.03 ± 1.22	**13.55** **±** **0.44**	>80	31.55 ± 2.28
Degree of resistance^*∗*^	0.84	1.19	>1.72	1.13	1.37	0.83	>1.16	0.65
MDA-MB-231-*pcDNA*	**19.87** **±** **1.77**	23.12 ± 1.65	72.23 ± 4.54	**20.42** **±** **1.71**	28.32 ± 1.57	**18.13** **±** **2.08**	68.91 ± 6.30	48.12 ± 1.99
MDA-MB-231-*BCRP*	**18.22** **±** **0.81**	26.42 ± 3.01	63.16 ± 3.30	**19.89** **±** **0.62**	24.41 ± 1.77	**17.66** **±** **1.11**	74.32 ± 3.92	37.23 ± 4.51
Degree of resistance	0.92	1.14	0.87	0.97	0.86	0.97	1.08	0.77
HCT116 (*p53*^*+/+*^)	26.75 ± 1.23	34.52 ± 1.74	59.10 ± 3.06	33.44 ± 3.62	30.76 ± 1.61	28.43 ± 3.01	>80	31.19 ± 1.26
HCT116 (*p53*^−^/^−^)	**17.44** **±** **2.01**	41.17 ± 3.14	>80	24.58 ± 3.48	33.63 ± 3.77	28.09 ± 1.75	>80	29.52 ± 0.98
Degree of resistance	0.65	1.19		0.74	1.09	0.99		0.95
U87MG	**12.42** **±** **0.66**	**18.03** **±** **1.77**	47.84 ± 4.36	**18.01** **±** **1.19**	**16.13** **±** **2.04**	**18.44** **±** **2.11**	47.89 ± 2.24	46.55 ± 2.43
U87MG.Δ*EGFR*	**14.36** **±** **1.48**	**20.12** **±** **0.81**	59.60 ± 5.12	**13.92** **±** **2.07**	**18.72** **±** **1.45**	**15.52** **±** **0.88**	55.20 ± 4.79	28.30 ± 1.11
Degree of resistance	1.16	1.12	1.25	0.77	1.16	0.84	1.15	0.60
HepG2	**17.45** **±** **2.05**	29.17 ± 1.30	>80	**18.75** **±** **1.41**	25.88 ± 1.56	**14.95** **±** **1.34**	>80	69.48 ± 2.93
AML12	42.09 ± 1.17	>80	>80	61.34 ± 3.39	56.12 ± 2.67	65.17 ± 4.31	>80	>80
Selectivity index^*∗∗*^	2.41	>2.74		3.27	2.17	4.56		1.15

(^*∗*^): the degree of resistance was determined as the ratio of the IC_50_ value in the resistant divided by the IC_50_ in the sensitive cell line; CEM/ADR5000, MDA-MB-231-*BCRP,* HCT116 (*p53*^−^/^−^), and U87MG.Δ*EGFR* were used as the corresponding resistant counterpart for CCRF-CEM, MDA-MB-231-*pcDNA*, HCT116 (*p53*^*+/+*^), and U87MG, respectively; (^*∗∗*^): the selectivity index was determined as the ratio of the IC_50_ value in the normal AML12 hepatocytes divided by the IC_50_ in HepG2 hepatocarcinoma cells; in bold: significant cytotoxic effect [7, 39, 40]; nd: not determined; HRB: crude methanol extract from the bark of *Hypericum roeperianum*, HRBa: portion obtained by exhaustion of HRB with ethyl acetate; HRBa1-5: fractions from HRBa; HRBb: residual fraction obtained after exhaustion of HRB with ethyl acetate. The data for doxorubicin used as positive control in similar experimental conditions are shown in Table 2.

**Table 2:** Recorded IC_50_ values following RRA for phytochemicals isolated from the bark of *Hypericum roeperianum* and reference drug, doxorubicin, towards drug-sensitive, MDR cancer cells lines and hepatocytes after 72 h incubation.

Cell lines	Samples, IC_50_ values in *µ*M, and degrees of resistance^*∗*^ or selectivity index^*∗∗*^						
	1	3	4	5	7	8	Doxorubicin
CCRF-CEM	14.44 ± 0.53	16.80 ± 0.96	19.94 ± 2.12	38.58 ± 2.11	23.28 ± 1.46	16.31 ± 2.12	**0.02** **±** **0.00**
CEM/ADR5000	18.27 ± 1.56	52.95 ± 3.08	23.21 ± 1.66	38.46 ± 4.07	54.04 ± 4.38	43.47 ± 2.97	122.96 ± 10.94
Degree of resistance^*∗*^	1.26	3.15	1.16	1.00	2.32	2.66	6,683.00
MDA-MB-231-*pcDNA*	16.47 ± 0.74	43.80 ± 3.47	>153.85	75.15 ± 4.88	20.73 ± 1.32	36.89 ± 2.73	**0.13** **±** **0.01**
MDA-MB-231-*BCRP*	14.95 ± 1.17	33.60 ± 1.99	20.38 ± 1.17	62.94 ± 5.32	22.16 ± 2.88	30.50 ± 1.88	**0.79** **±** **0.08**
Degree of resistance	0.91	0.77	<0.13	0.84	1.07	0.83	6.14
HCT116 (*p53*^*+/+*^)	17.36 ± 1.84	46.67 ± 3.38	40.17 ± 3.09	75.48 ± 6.10	>91.74	37.79 ± 2.92	**0.48** **±** **0.06**
HCT116 (*p53*^−^/^−^)	44.20 ± 3.21	>165.29	>153.85	112.27 ± 8.49	>91.74	>85.47	**1.78** **±** **0.08**
Degree of resistance	2.55	>3.54	>6.15	1.49	nd	>2.26	3.73
U87MG	16.16 ± 1.09	74.44 ± 4.75	106.00 ± 6.74	61.42 ± 3.39	29.70 ± 1.77	35.50 ± 3.28	**0.26** **±** **0.03**
U87MG.Δ*EGFR*	14.69 ± 1.55	44.98 ± 5.22	30.37 ± 2.91	59.04 ± 6.01	12.72 ± 0.75	30.61 ± 3.14	**0.98** **±** **0.07**
Degree of resistance	0.91	0.60	0.29	0.96	0.43	0.86	3.79
HepG2	21.68 ± 3.18	44.21 ± 2.65	32.40 ± 3.72	64.73 ± 5.77	25.19 ± 1.69	31.29 ± 1.19	**4.56** **±** **0.48**
AML12	47.34 ± 0.81	>165.29	45.35 ± 3.52	150.02 ± 7.03	20.89 ± 1.17	>85.47	52.90 ± 4.09
Selectivity index^*∗∗*^	2.18	>3.74	1.40	2.32	0.83	>2.73	11.59

(^*∗*^): the degree of resistance was determined as the ratio of the IC_50_ value in the resistant divided by the IC_50_ in the sensitive cell line; CEM/ADR5000, MDA-MB-231-*BCRP,* HCT116 (*p53*^−^/^−^), and U87MG.Δ*EGFR* were used as the corresponding resistant counterparts for CCRF-CEM, MDA-MB-231-*pcDNA*, HCT116 (*p53*^*+/+*^), and U87MG, respectively; (^*∗∗*^): the selectivity index was determined as the ratio of the IC_50_ value in the normal AML12 hepatocytes divided by the IC_50_ in HepG2 hepatocarcinoma cells; in bold: significant cytotoxic effect [7, 39, 40], the cytotoxicity of compound **6** (betulenic acid) on these cell lines was previous reported [41], and this compound was no more tested in this study, no IC_50_ value was recorded at up to 100 *µ*M with compound **2**; nd: not determined; **1:** trichadonic acid; **3:** 2-hydroxy-5-methoxyxanthone; **4:** 1,3,6,7-tetrahydroxyxanthone or norathyriol; **5:** 1,3,5,6-tetrahydroxyxanthone; **7:** 3′-hydroxymethyl-2′-(4″-hydroxy-3″,5″-dimethoxyphenyl)-5′,6′:5,6-(6,8-dihydroxyxanthone)-1′,4′-dioxane; and **8:** 3′-hydroxymethyl-2′-(4″-hydroxy-3″,5″-dimethoxyphenyl)-5′,6′:5,6-(xanthone)-1′,4′-dioxane.

## Data Availability

All data generated or analyzed during this study are included in this published article and its supplementary information files.

## Abbreviations

**1**: Trichadonic acid
**2**: Fridelan-3-one
**3**: 2-Hydroxy-5-methoxyxanthone
**4**: 1,3,6,7-Tetrahydroxyxanthone or norathyriol
**5**: 1,3,5,6-Tetrahydroxyxanthone
**6**: Betulenic acid
**7**: 3′-Hydroxymethyl-2′-(4″-hydroxy-3″,5″-dimethoxyphenyl)-5′,6′:5,6-(6,8-dihydroxyxanthone)-1′,4′-dioxane
**8**: 3′-Hydroxymethyl-2′-(4″-hydroxy-3″,5″-dimethoxyphenyl)-5′,6′:5,6-(xanthone)-1′,4′-dioxane; ABC, ATP-binding cassette; BCRP, breast cancer resistance protein; DMSO, dimethylsulfoxide; D.R., resistance; EGFR, epidermal growth factor receptor; FITC, flourescein isothiocynate; H_2_O_2_, hydrogen peroxide; H2DCFH-DA, 2′,7′-dichlorodihydrofluoresceine diacetate; HRB, methanol extract of the bark of *Hypericum roeperianum*; HRBa, portion obtained by exhaustion of HRB with ethyl acetate; HRBa1-5, fractions from HRBa; HRBb, Residual fraction obtained after exhaustion of HRB with ethyl acetate; IC_50,_ 50% inhibitory concentration; MDR, multidrug resistance; PBS, phosphate buffer saline; P-gp, P-glycoprotein; PI, propidium iodide; and ROS, reactive oxygen species

## Supplementary Materials

Supplementary Materials Additional File: S1: NMR spectra and main chemical shifts of compounds: NMR spectra and main chemical shifts of the isolated compounds; S2: diagrams of annexin V/PI staining of CCRF-CEM with HRB and trichadonic acid.
