# Supplementary material for: Cytotoxic Constituents of the Bark of Hypericum roeperianum towards Multidrug-Resistant Cancer Cells
Source: Evid Based Complement Alternat Med. 2020 Sep 25;2020:4314807. doi: 10.1155/2020/4314807 (PMC7532997; doi:10.1155/2020/4314807)
Supplement: Supplementary Materials — Additional File: S1: NMR spectra and main chemical shifts of compounds: NMR spectra and main chemical shifts of the isolated compounds; S2: diagrams of annexin V/PI staining of CCRF-CEM with HRB and trichadonic acid. [file 4314807.f1.docx]

Cytotoxicity of trichadonic acid and other phytochemicals from the bark of *Hypericum roeperianum* towards multifactorial drug resistant cancer cells

Simplice Beaudelaire Tankeo^a^, Francois Damen^b^, Michel-Gael F. Guefack^a^, Armelle T. Mbaveng^a^, Gabin T. M. Bitchagno^b^, İlhami Çelik^c^, Victor Kuete^a*^

*^a^Department of Biochemistry, Faculty of Science, University of Dschang, P.O. Box 67, Dschang, Cameroon;*

*^b^Department of Chemistry, Faculty of Science, University of Dschang, P.O. Box 67, Dschang, Cameroon*

*^c^Department of Chemistry*, *Faculty of Science, Eskisehir Technical University, 26470 Eskisehir, Turkey*

*****Corresponding author:**

*Tel: +237 677355927; E-mail:* [*kuetevictor@yahoo.fr*](mailto:kuetevictor@yahoo.fr)*; P.O. Box 1499 Bafoussam, Cameroon (Prof. Dr. Victor Kuete)*

*S1. NMR spectra and main chemical shifts of compounds*

The NMR spectra as well as the main chemical shifts of the isolated compounds are given below.


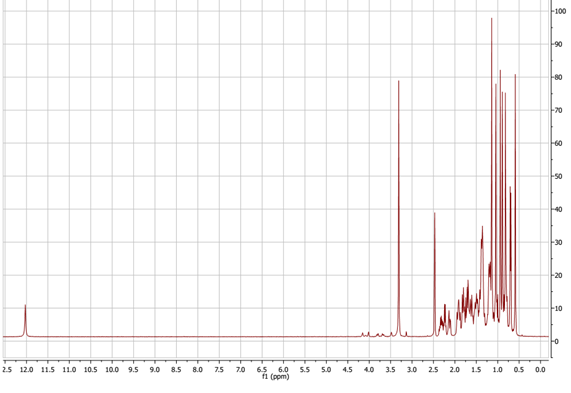


^1^H NMR (400 MHz, DMSO-*d_6_*) of compound


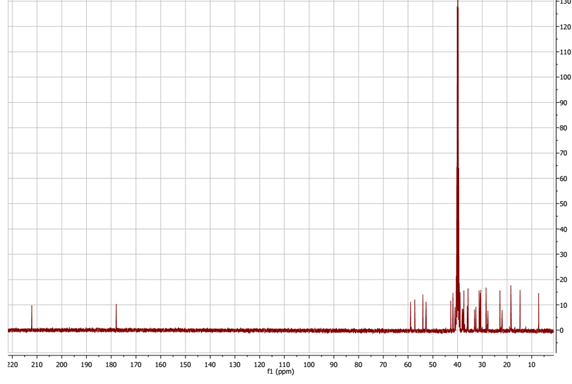


^13^C NMR (100 MHz, DMSO-*d_6_*) of compound

Trichadonic acid (**1**) : White powder ; ^13^C NMR (125 MHz, CDCl_3_) *δc* 22.0 (C-1), 14.7 (C-24), 18,5 (C-7), 18,4(C-25), 7,2 (C-23), 22,9 (C-26), 27,7 (C-12), 28,5 (C-20), 30,6 (C-29), 30,8 (C-17), 31,3 (C-28), 32,9 (C-15), 32,9 (C-21), 33,2 (C-30), 35,8 (C-19), 36,1 (C-16), 37,4 (C-22), 37,8 (C-11), 38,1 (C-9), 38,9 (C-14), 41,0 (C-6), 41,1 (C-2), 41,8 (C-5), 42,7 (C-18), 52,7 (C-8), 54,0 (C-13), 57,3 (C-4), 59 ,0 (C-10), 178,0 (C-27), 212,1 (C-3) [1].


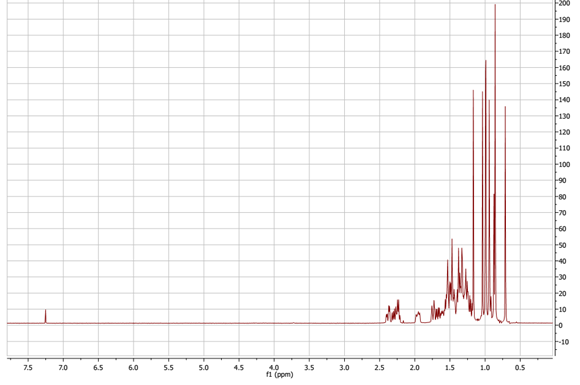


^1^H NMR (500 MHz, CDCl_3_) of compound


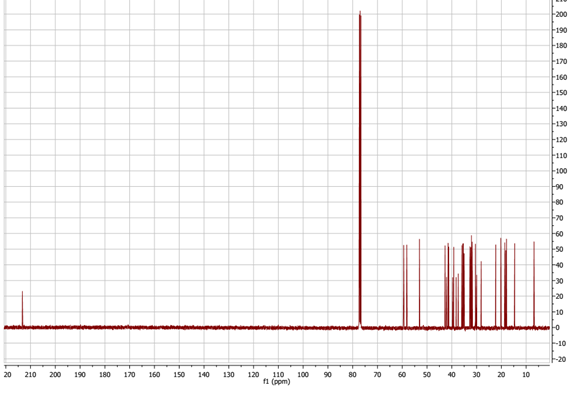


^13^C NMR (125 MHz, CDCl_3_) of compound

Fridelan-3-one (**2**) : White powder ; ^13^C NMR (100 MHz, CDCl_3_) *δc* 22.3 (C-1), 41.7 (C-2), 213.3 (C-3), 58.2 (C-4), 42.1 (C-5), 41.5 (C-6), 18.2 (C-7), 53.1 (C-8), 37.4 (C-9), 59.4 (C-10), 36.0 (C-11), 30.5 (C-12), 39.7 (C-13), 38.3 (C-14), 32.4 (C-15), 36.0 (C-16), 30.0 (C-17), 42.8 (C-18), 35.3 (C-19), 28.2 (C-20), 32.7 (C-21), 39.2 (C-22), 6.8 (C-23), 14.6 (C-24), 17.9 (C-25), 20.3 (C-26), 18.7 (C-27), 32.1 (C-28), 35.0 (C-29), 31.8 (C-30) [1].


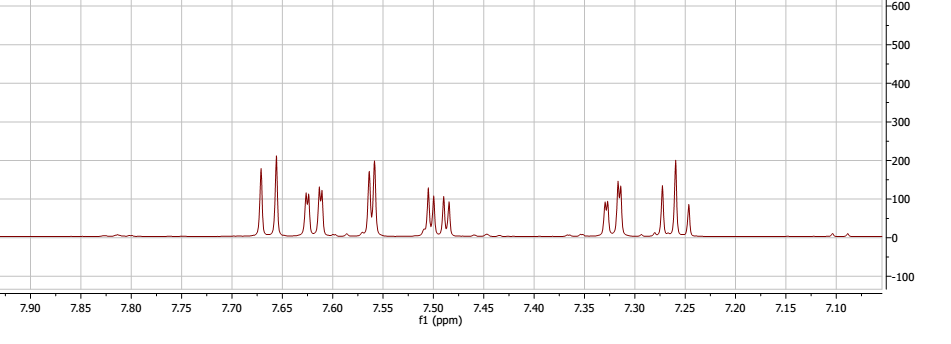


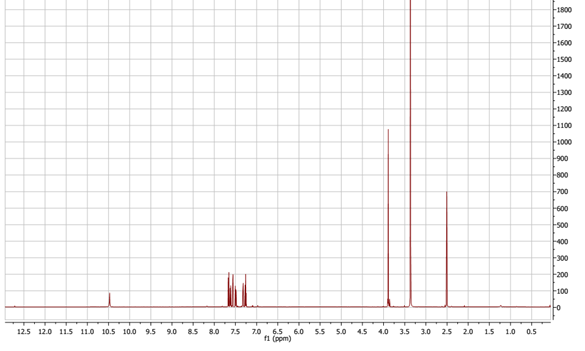


^1^H NMR (400 MHz, DMSO-*d_6_*) of compound


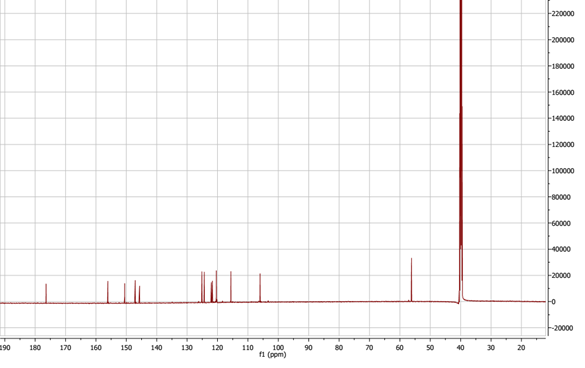


^13^C NMR (100 MHz, DMSO-*d_6_*) of compound

2-Hydroxy-5-methoxyxanthone (**3**): yellow powder ; ^13^C NMR (100 MHz, DMSO-*d_6_*) *δc* 106.0 (C-1), 121.7 (C-1a), 156.1 (C-2), 125.2 (C-3), 120.4 (C-4), 150.6 (C-4a), 147.1 (C-5), 145.6 (C-5a), 120.3 (C-6), 124.4 (C-7), 115.6 (C-8), 122.1 (C-8a), 176.4 (C-9), 56.2 (C-1) [2].


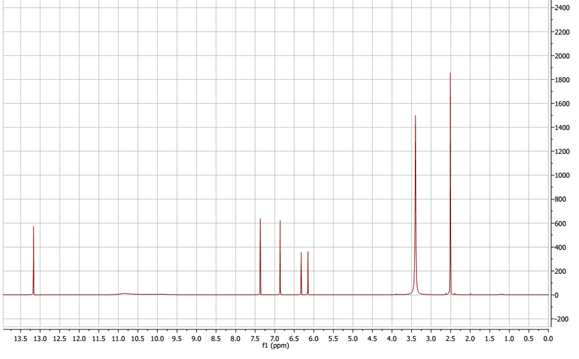


^1^H NMR (400 MHz, DMSO-*d_6_*) of compound

6.29(1H, d), 6.11(1H, d), 6.82(1H,d), 7.33(1H, d), 13.1 s


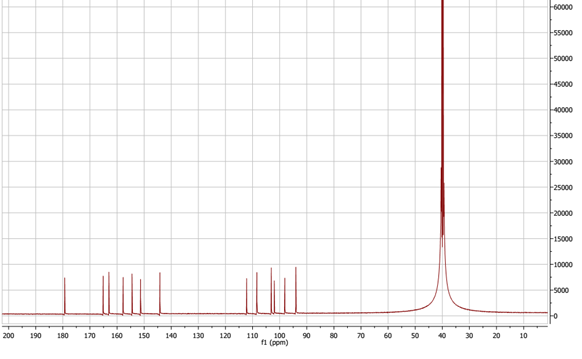


^13^C NMR (100 MHz, DMSO-*d_6_*) of compound

1,3,6,7-Tetrahydroxyxanthone or norathyriol (**4**): green-yellowsh powder ; ^13^C NMR (125 MHz, DMSO-*d_6_*)*, δc* 165.1 (C-1), 102.0 (C-1a), 94.0 (C-2), 163.0 (C-3), 98.1 (C-4), 157.7 (C-4a), 154.6 (C-5), 151.3 (C-5a), 103.0 (C-6), 144.2 (C-7), 108.3 (C-8), 112.1 (C-8a), 179.3 (C-9) [3].


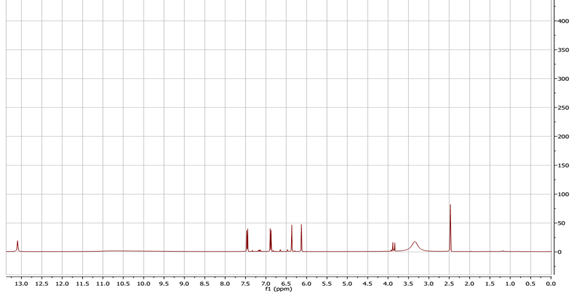


^1^H NMR (400 MHz, DMSO-*d_6_*) of compound


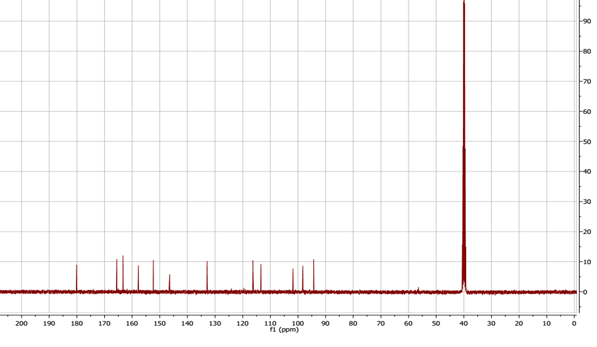


^13^C NMR (100 MHz, DMSO-*d_6_*) of compound

1,3,5,6-tetrahydroxyxanthone (**5**) : yellow powder ; ^13^C NMR (100 MHz, DMSO-*d_6_*) *δc* 165.1 (C-1), 102.0 (C-1a), 94.0 (C-2), 163.0 (C-3), 98.1 (C-4), 157.7 (C-4a), 154.6 (C-5), 151.3 (C-5a), 103.0 (C-6), 144.2 (C-7), 108.3 (C-8), 112.1 (C-8a), 179.3 (C-9) [4].


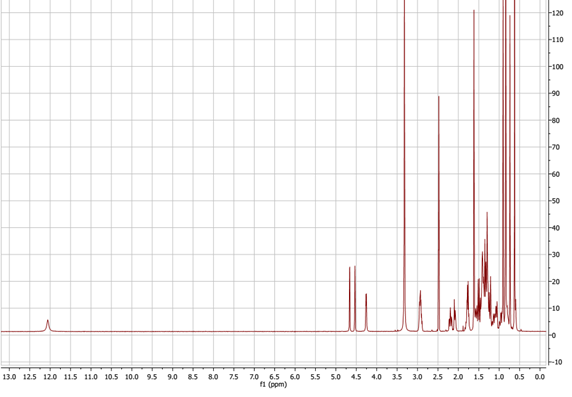


^1^H NMR (400 MHz, DMSO-*d_6_*) of compound


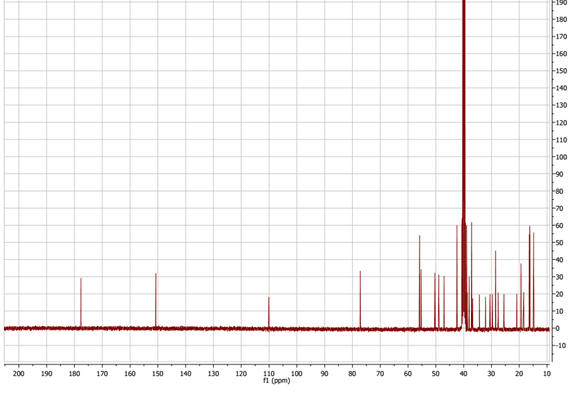


^13^C NMR (100 MHz, DMSO-*d_6_*) of compound

Betulenic acid (**6**): White powder ; ^13^C NMR (100 MHz, DMSO-*d_6_*) *δc* 38.1 (C-1), 30.8 (C-2), 79.7 (C-3), 40.1 (C-4), 56.9 (C-5), 19.4 (C-6), 35.6 (C-7), 42.0 (C-8), 52.0 (C-9), 39.7 (C-10), 22.1 (C-11), 26.9 (C-12), 38.9 (C-13), 43.9 (C-14), 31.7 (C-15), 33.4 (C-16), 57.5 (C-17), 50.0 (C-18), 50.1 (C-19), 152.0 (C-20), 28.6 (C-21), 38.4 (C-22), 28.1 (C-23), 16.1 (C-24), 16.6 (C-25), 16.9 (C-26), 15.1 (C-27), 180.0 (C-28), 110.1 (C-29), 19.5 (C-30) [1].


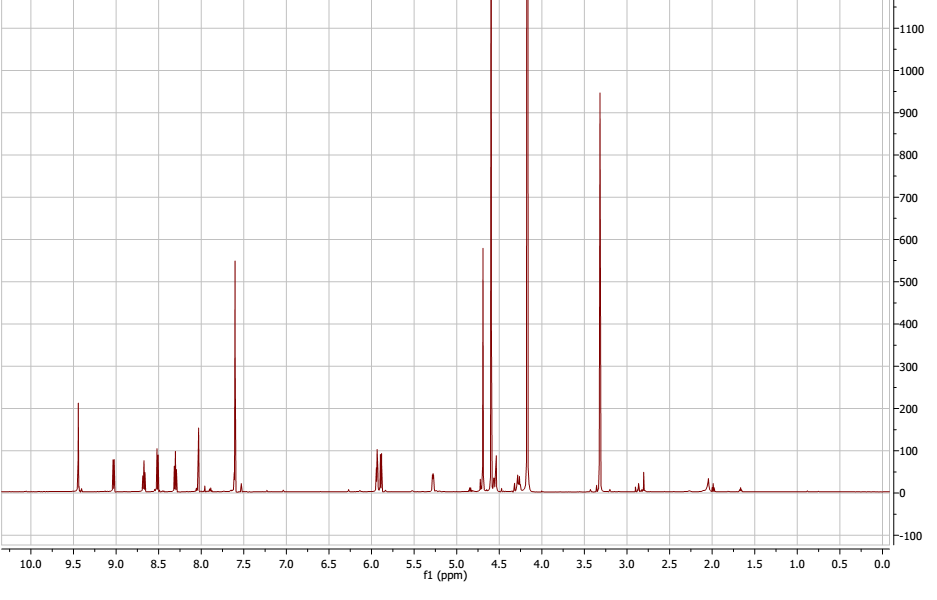


^1^H NMR (400 MHz, DMSO-*d_6_*) of compound


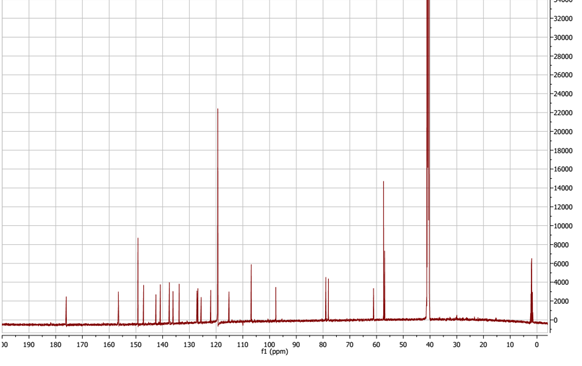


^13^C NMR (100 MHz, DMSO-*d_6_*) of compound

3′-Hydroxymethyl-2′-(4′′-hydroxy-3′′,5′′-dimethoxyphenyl)-5′,6′:5,6-(6,8-dihydroxyxanthone)-1′,4′-dioxane (**7**) : yellow powder ; ^13^C NMR (100 MHz, DMSO-*d_6_*) *δc* 97.7 (C-1), 115,2 (C-1a), 147,1 (C-2), 140,9 (C-3), 133,8 (C-4), 142,5 (C-4a), 119,4 (C-5), 136,1 (C-5a), 156,6 (C-6), 125,6 (C-7), 127,2 (C-8), 122,0 (C-8a), 176,0 (C-9), 126,8 (C-1’), 106,9 (C-2’), 149,2 (C-3’), 137,5(C-4’), 149,2 (C-5’), 106,9 (C-6’), 78,0 (C-7’), 79,0 (C-8’), 61,1 (C-9’), 57,4 (3’5’-OMe), 57,0 (1-OMe) [5, 6].


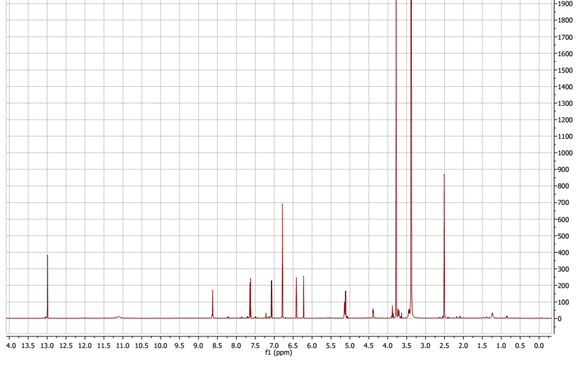


^1^H NMR (400 MHz, DMSO-*d_6_*) of compound


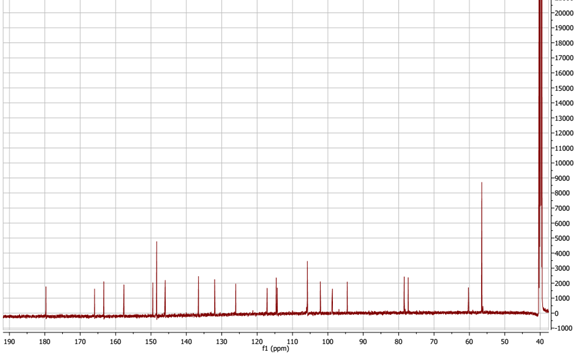


^13^C NMR (100 MHz, DMSO-*d_6_*) of compound

3′-Hydroxymethyl-2′-(4′′-hydroxy-3′′,5′′-dimethoxyphenyl)-5′,6′:5,6-(xanthone)-1′,4′-dioxane (**8**): yellow powder ; ^13^C NMR (100 MHz, DMSO-*d6*) *δc* 117.2 (C-1), 132.0 (C-1a), 114.6 (C-2), 146.0 (C-3), 148.4 (C-4), 149.5 (C-4a), 98.7 (C-5), 157.7 (C-5a), 165.6 (C-6), 94.5 (C-7), 163.3 (C-8), 102.2 (C-8a), 179.7 (C-9), 126.0 (C-1’), 106.8 (C-2’), 148.4 (C-3’), 136.6 (C-4’), 148.4 (C-5’), 106.8 (C-6’), 77.3 (C-7’), 78.4 (C-8’), 60.3 (C-9’), 56.5 (C-3’/5’-OMe)

*S2. Diagrams of annexin V/PI staining of CCRF-CEM with HRB and trichadonic acid*

**Figure S1.** Apoptosis induced by the crude extract (HRB), trichadonic acid and doxorubicin after 24 h on CCRF-CEM leukemia cells as determined by annexin V/PI assay. Apoptosis was assessed by flow cytometry after annexin V-PI double staining. IC_50_ values were 13.71 µg/mL for HRB, 14.44 µM for trichadonic acid and 0.02 µM for Doxorubicin. Necrotic cells lose membrane integrity, allowing PI entry. Q2-LL: viable cells exhibit annexin V (-)/PI (-); Q2-LR: early apoptotic cells exhibit annexin (+)/PI (-); Q2-UR and Q2-UL: late apoptotic cells or necrotic cells exhibit annexin V (+)/PI (+) or annexin V (-)/PI (+).

**References**

1. Mahato SB, Kundu SP: **13C NMR spectra of pentacyclic triterpenoids-A compilation and some salient features.** *Phytochemistry* 1994, **37**:1517-1575.

2. Cardona ML, Pedro JR, Seoane E, Vidal R: **Xanthone constituents of *Hypericum canariensis***. *J Nat Prod* 1985, **48**(3):467-469.

3. Çirak C: **Hypericin in *Hypericum lydium* Boiss. growing in Turkey**. *Biochem Syst Ecol* 2006, **34**(12):897-899.

4. Don MJ, Huang YJ, Huang RL, Lin YL: **New phenolic principles from *Hypericum sampsonii***. *Chemical & pharmaceutical bulletin* 2004, **52**(7):866-869.

5. Abou-shoer M, Habib A-A, Chang C-J, Cassady JM: **Seven xanthonolignoids from *Psorospermum febrifugum***. *Phytochemistry* 1989, **28**(9):2483-2487.

6. Cardona ML, Fernández MI, Pedro JR, Seoane E, Vidal R: **Additional new xanthones and xanthonolignoids from *Hypericum canariensis***. *J Nat Prod* 1986, **49**(1):95-100.
